# Comparing Approaches to Link SF-36 PF-10 Scores to PROMIS Physical Function: A Validation Study in Three Clinical Samples

**DOI:** 10.1007/s11606-025-09496-5

**Published:** 2025-04-29

**Authors:** Audrey Yuki Brinker, Sandra Nolte, Felix H. Fischer, Alexander Obbarius, Matthias Rose, Gregor Liegl

**Affiliations:** 1 Center for Patient-Centered Outcomes Research (CPCOR), Department of Psychosomatic Medicine, Charité – Universitätsmedizin Berlin, Corporate Member of Freie Universität Berlin, Humboldt-Universität Zu Berlin, and Berlin Institute of Health, Berlin, Germany; 2 Person-Centred Research, Eastern Health Clinical School, Monash University, Melbourne, VIC Australia; 3 School of Health Sciences, Swinburne University of Technology, Melbourne, VIC Australia

**Keywords:** quality of life, patient-reported outcome, PROMIS, linking, SF-36

## Abstract

**Background:**

Physical function (PF) is a central patient-reported outcome (PRO) in many clinical conditions. However, the variety of existing PRO measures (PROMs) yield scores on different scales, limiting the score comparability and interpretability. To overcome this gap, the Patient-Reported Outcomes Measurement Information System (PROMIS®) established a standardized T-score metric using item response theory (IRT). As such, different PROMs measuring PF can be *linked* to this common metric, allowing for efficient harmonization of scores. Linking algorithms allow conversion of SF-36 PF-10 scores to the PROMIS-PF metric, but these methods have not been validated in independent clinical samples.

**Objective:**

To validate and compare two established linking methods for the translation of SF-36 PF-10 scores to the PROMIS-PF metric in clinical populations.

**Design:**

Two previously proposed linking approaches were applied to estimate PROMIS-PF T-scores based on the SF-36 PF-10:

1. Item-level linking,
2. Cross-walk tables.

The directly observed T-scores from the 20-item PROMIS-PF short form (PROMIS-PF20a) served as a benchmark against which the linked T-scores from the SF-36 PF-10 were compared. Results were compared to a newly estimated IRT-model based on the study’s dataset.

**Participants:**

Patients from cardiology (*n* = 185), rheumatology (*n* = 172), and psychosomatic medicine (*n* = 262), who completed both the PROMIS-PF20a and the SF-36 PF-10.

**Main Measures:**

PROMIS-PF20a, SF-36 PF-10.

**Key Results:**

All linking approaches demonstrated high association with observed PROMIS-PF20a T-scores (Pearson correlation ≥ 0.84) and indicated negligible practical differences at the group level (standardized mean difference < 0.2).

**Conclusions:**

Two currently available linking approaches can reliably translate SF-36 PF-10 scores to standardized PROMIS-PF T-scores across different clinical samples, eliminating the need for re-estimating models in new datasets. As all linking algorithms ultimately presented highly comparable results, cross-walk tables may be preferred as the most practicable approach, allowing for score conversion without complex statistical modeling.

**Supplementary Information:**

The online version contains supplementary material available at 10.1007/s11606-025-09496-5.

## INTRODUCTION

Assessing patient-reported outcomes (PROs) is essential to patient-centered care, as it provides direct insight into patient’s functional status, well-being, and quality of life.^1,2^ Among PROs, physical function (PF) is a particularly important health domain,^3–5^ referring to the individual’s ability to perform daily activities, maintain independence, and engage in meaningful social and occupational roles^6^. Therefore, PF is frequently assessed in chronic diseases, such as cardiovascular diseases^7,8^ and rheumatic diseases,^9,10^ as well as in several psychosomatic conditions.^11^

To date, a vast number of PRO measures (PROMs) for PF have been developed, widely differing in format, length, response options, recall period, scoring, benchmarks, and psychometric properties.^12^ This heterogeneity complicates the comparability of scores across studies, clinical settings, and healthcare systems. This limits not only the comparison of results from cross-national data or across clinical groups but even restricts comparisons of the same outcomes and for the same illness.^12,13^ One approach to standardize PRO assessment is the development of generic PROMs, which aim to measure the key dimensions of PROs consistently across different populations. As a prominent example of such a generic PROM, the 10-item PF scale from the Medical Outcome Study Short Form- 36 Survey (SF-36 PF-10) is widely accepted and implemented in practice.^14^ However, conventional PROMs, including the SF-36 PF-10, are often a compromise between practicability and measurement precision, and they remain difficult to directly compare with other measures, as they do not use a standardized metric.^12,13^

To overcome these gaps, advanced psychometric approaches within the framework of item response theory (IRT) can be utilized to efficiently harmonize PROMs. IRT is a modern measurement approach that models the relationship between individual item responses and the underlying construct being measured. It does this by estimating *item parameters*, which allow each item’s response pattern to provide meaningful information about a person’s position on the construct. These parameters can be used for the *linking*, which refers to the psychometric process of establishing a relationship between different PROMs that measure the same construct, allowing scores from one instrument to be mapped onto to the scale of another.^12,13^ The Patient-Reported Outcomes Measurement Information System (PROMIS®), funded by the U.S. National Institutes of Health, provides standardized metrics for several generic PRO domains, including PF.^13,15^ Prior research has developed two linking approaches to convert SF-36 PF-10 scores to the PROMIS-PF metric:

1. Item-level linking, which uses item parameters from the original PROMIS IRT-model
2. Cross-walk tables, which provide a simplified score conversion method without requiring specialized statistical expertise and software. Currently, the PROsetta Stone project enables for a free access to such tables for multiple instruments through its website (www.prosettastone.org)

While both linking algorithms have been carefully developed in the past and contribute significantly to the standardization of PROs, their applicability to new data remains unexplored.^16,17^ Validity is contextual and it is unclear whether previously established linking relationships hold across different patient populations.^18–20^ Validation of the linking relationship in different clinical samples is required to enhance generalizability.^16,17^ Furthermore, practical guidance on which linking approach is most suitable for clinical use is lacking.

As one of the first in the field, this study evaluates and compares two previously developed algorithms to link SF-36 PF-10 scores to the PROMIS-PF metric in three independent clinical samples in Germany. We applied both item-level linking and cross-walk linking, and compared the resulting T-score estimates to observed PROMIS-PF20a T-scores. In addition, we evaluated a newly estimated IRT-model based on the study’s dataset. By examining the agreement between these methods, we aim to determine whether existing linking approaches can be reliably applied to clinical populations without requiring re-estimation. Our findings will help guide researchers and clinicians in selecting the most practical and accurate approach for integrating SF-36 PF-10 and PROMIS-PF scores in clinical research and practice.

## METHOD

### Measures

#### Medical Outcomes Study Short Form 36 Physical Function Scale (SF-36 PF-10)

The SF-36 PF-10 is internationally established and is one of the most frequently used conventional measures of generic self-reported PF.^14^ It consists of 10 items covering physical activities at various levels of difficulty. Respondents rate the items on a 3-point scale with higher scores indicating better PF due to fewer limitations, yielding raw sum scores ranging from 10 to 30. In this study, we used the German version of the SF-36 PF-10, which has been validated with robust psychometric evidence.^21,22^

#### The PROMIS-PF Metric

The PROMIS-PF item bank defines PF as the ability to carry out activities that require physical capability, ranging from self-care to more-vigorous activities.^6,16^ PROMIS-PF items assess a large range of PF and target the subdomains mobility, dexterity, central regions, and complex activities that involve more than one subdomain.^16^ Accordingly, the full PROMIS-PF item bank covers a wide range of items, yet can also be condensed in short forms. For instance, the generic PROMIS-PF fixed-length 20-item short form (PROMIS-PF20a) was derived from the PROMIS-PF item bank using IRT and is constructed to cover the full range of the PF trait.^23,24^ The corresponding response options of the PROMIS items are provided on a 5-point Likert scale with higher scores indicating better functioning. The resulting scores are linearly transformed to the PROMIS T-score metric, which is a standardized metric based on a normal distribution with a general population mean of 50 and a standard deviation (SD) of 10. A T-score of 50 represents the mean PROMIS T-score, matching the marginal distributions of gender, age, race, and education on the 2000 U.S. Census^16^. T-scores above 50 indicates above-average PF, while T-scores below 50 indicate below-average functioning levels.

Good psychometric properties of different PROMIS-PF measures, including the PROMIS-PF20a, have been demonstrated for different languages with cultural adaptations extending beyond the USA.^11,25–27^ Among others, the PROMIS-PF item bank was translated and cross-culturally adapted for use in German-speaking populations.^11,19^ In this study, the German version of the PROMIS-PF20a was utilized to generate PROMIS-PF T-scores.

### Data Collection

Through secondary data analysis, we investigated three clinically diverse samples of adult patients that had been collected as part of two earlier studies.^11,28^ Participants were recruited from the Clinic for Cardiology and Angiology (cardiology sample) and the Department of Rheumatology and Clinical Immunology (rheumatology sample) at Charité—Universitätsmedizin Berlin, Germany.^28^ Additionally, data from the outpatient clinic of the Department of Psychosomatic Medicine (psychosomatic sample) at Charité was collected.^11^ The psychosomatic sample was clinically diverse including patients with somatoform disorders, chronic pain, eating disorders, and patients with a variety of physical conditions associated with mental disorders and psychological distress.^11^ Both PROMIS-PF20a and the SF-36 PF-10 were administered to the samples either as paper-based questionnaires or electronically using personal digital assistant devices.^11,28^

### Statistical Analysis

Before conducting the linking of SF-36 PF-10 scores to the PROMIS-PF metric, the assumptions of IRT-based linking were examined, following the PROMIS analysis plan.^29^ Subsequently, linking was performed by estimating the PROMIS-PF T-scores based on the SF-36 PF-10 scores, utilizing the proposed methods.^16,17^ (S1 Figure). Finally, the degree of agreement between observed and linked PROMIS T-scores was assessed.

#### Assumptions of IRT-Based Linking

As a key assumption of unidimensional IRT-based linking, the unidimensionality of the pooled set of items from both PROMs was evaluated. To test whether the items of both the SF-36 PF-10 and PROMIS-PF20a measure the same underlying construct, a confirmatory factor analysis (CFA) was conducted using a one-factor model with robust weighted least squares means and variance adjusted estimator (WLSMV).^29^ Additionally, an exploratory bi-factor model was conducted to not only control for a shared general factor, but also for specific group factors in form of subdomains.^29^ Benchmark values were set according to the recommendations outlined in the PROMIS scientific standards document (https://www.healthmeasures.net/images/PROMIS/PROMISStandards_Vers2.0_Final.pdf).

Due to possible variations in results for age, gender, and the clinical samples, measurement invariance was examined by testing for differential item functioning (DIF).^28^ For this, the Nagelkerke R2 coefficient was used, with a change > 0.03 indicating potentially relevant DIF.^16^

#### T-Score Linking

To convert the SF-36 PF-10 scores to PROMIS T-scores, we applied two currently available linking approaches, which we compared to a third approach based on a newly estimated IRT-model (S1 Figure):

1. The first linking approach is based on the original common IRT-model estimated in the PROMIS Wave 1 sample. In the original calibration of the PROMIS-PF metric, the items of the SF-36 PF-10 were included alongside the newly established PROMIS-PF items. Consequently, the item parameters from this original IRT-model can be directly used to estimate PROMIS-PF T-scores based on the SF-36 PF-10 (“item-level linking”).^16^
2. As a second approach, the cross-walk table by Schalet et al. was used for simple translation of raw SF-36 PF-10 scores to PROMIS-PF T-scores without the need for statistical expertise or specialized software. Individual item parameters are not necessary for this approach. This table was created using data from a subsample of the PROMIS Wave 1 study, where participants answered both the SF-36 PF-10 and selected PROMIS-PF items.^17^ In this process, the PROMIS-PF item parameters were fixed to the original metric,^16^ while the item parameters of the SF-36 PF-10 items were freely estimated.^17^ The resulting parameters were then used to create a user-friendly score cross-walk table (“cross-walk table”).
3. As a third approach, a new IRT-model was estimated through a fixed-parameter calibration, where the PROMIS-PF20a parameters were fixed to the original metric^16^ and the SF-36 PF-10 parameters were re-estimated based on the sample from this study (“IRT re-estimation”).

The T-scores resulting from the PROMIS-PF20a served as the benchmark against which the linked SF-36 PF-10 T-scores were compared.

#### Evaluation of Agreement of Linking Across Linking Approaches

Agreement between linked SF-36 PF-10 T-scores and observed PROMIS-PF20a T-scores was evaluated for each linking approach, both in the full analytic sample and within each clinical sample separately.

PROMIS-PF20a T-scores and each of the linked SF-36 PF-10 T-scores were initially investigated with regard to their correlation and mean differences (SD) to descriptively assess their associations and agreement.^12,29^

To quantify the accuracy of estimates, mean absolute errors (MAEs) and root mean square errors (RMSEs) were investigated between scores estimated from PROMIS-PF20a and SF-36 PF-10^12,30^. The 95% confidence intervals (CI) to these single-point estimates were derived through Bootstrap.^31^ Moreover, to quantify agreement on the group level, standardized mean differences (SMD) for paired samples along with 95% CI were calculated,^12^ with |SMD| of < 0.2 interpreted as a negligible effect size.^32^

Bland–Altman plots were conducted to illustrate the agreement of the linking approaches for each clinical sample at the individual level of the patients.^12,33^

R version 4.3.1 was used for all statistical analyses, including the R packages effsize, lavaan, lordif, mirt, and psych.^34–38^

## RESULTS

### Sample Characteristics

A total of *N* = 619 complete cases from cardiology (*n* = 185, 29.9%), rheumatology (*n* = 172, 27.8%), and psychosomatic medicine (*n* = 262, 42.3%) were included for analysis. From the initial sample, *n* = 48 were excluded due to entirely missing data in PROMIS-PF20a and/or SF-36 PF-10 (*n* = 5), or due to highly likely reversed coding bias (*n*= 43), implying an inconsistent response in the reversed-scored questionnaires, likely due to acquiescence, inattention, or confusion.^39^

The full analytic sample’s mean PF level, as measured by the PROMIS-PF20a T-score, was 42.6 ± 8.7, which is about ¾ SDs below the age- and gender-adjusted average general population PF score of Germany.^40^ The rheumatology sample showed the lowest PF level, while PROMIS-PF20a T-scores and SF-36 PF-10 sum scores were comparable between the cardiology sample and the psychosomatic sample (Table 1).

**Table 1:** Sample Characteristics

Variables	Initial sample (*N* = 667)	Analytic sample (*N* = 619)	Cardiology sample (*N* = 185)	Rheumatology sample (*N* = 172)	Psychosomatic sample (*N* = 262)
**Demographics**					
Female; * n* (%)	375 (56.2)	353 (57.0)	72 (38.9)	121 (70.3)	160 (61.1)
Mean age (SD)	52.4 (17.6)	51.8 (17.6)	64.2 (14.6)	52.4 (16.6)	42.6 (14.6)
**Clinical sample**					
Cardiology; * n* (%)	201 (30.1)	185 (29.9)	185 (100)	-	-
Rheumatology; *n* (%)	200 (30.0)	172 (27.8)	-	172 (100)	-
Psychosomatic; *n* (%)	266 (39.9)	262 (42.3)	-	-	262 (100)
**Chronic condition**					
Any; * n* (%)	599 (89.8)	558 (90.1)	168 (90.8)	157 (91.3)	233 (88.9)
Rheumatism; *n* (%)	153 (22.9)	137 (22.1)	15 (8.1)	99 (57.6)	23 (8.8)
Back pain; * n* (%)	272 (40.8)	252 (40.7)	62 (33.5)	51 (29.7)	139 (53.1)
Cardiovascular conditions; * n* (%)	221 (33.1)	205 (33.1)	141 (76.2)	29 (16.9)	35 (13.4)
Respiratory illness; * n* (%)	98 (14.7)	91 (14.7)	28 (15.4)	26 (15.1)	37 (14.1)
Cancer; * n* (%)	27 (4.0)	21 (3.4)	8 (4.3)	3 (1.7)	10 (3.8)
Diabetes; * n* (%)	67 (10.0)	61 (9.9)	30 (16.2)	10 (5.8)	21 (8.0)
Stroke; * n* (%)	23 (3.4)	21 (3.4)	8 (4.3)	8 (4.7)	5 (1.9)
Other; * n* (%)	272 (40.8)	254 (41.0)	49 (26.5)	80 (46.5)	125 (47.7)
**Physical functioning level**					
PROMIS-PF20a; T-score (SD)	42.2 (8.8)	42.6 (8.7)	43.5 (7.8)	39.4 (8.9)	44.0 (8.6)
SF-36 PF-10; raw summed score (SD)	21.9 (6.1)	22.0 (6.1)	22.2 (5.7)	19.9 (6.3)	22.6 (5.9)

*Abbreviations*: *n*, number; *PF*, physical function; *PROMIS-PF20a*, 20-item short form of the Patient-Reported Outcomes Measurement Information System physical function item bank; *SF-36 PF-10*, 10-item Short Form of the Medical Outcomes Study 36 Physical Function Scale; *SD*, standard deviation

### Assumptions of IRT-Based Linking

Most fit indices resulting from the CFA and the exploratory bifactor analysis suggested a sufficiently unidimensional structure (Table 2).

**Table 2:** Psychometric Properties of the Combined Set of PROMIS-PF20a and SF-36 PF-10 Items (*n* = 619)

	Statistics/indices	Criterion	Software	Results
Confirmatory factor analysis (CFA)	CFI	> 0.95	Lavaan (R package)	0.99
	TLI	> 0.95		0.99
	RMSEA	< 0.06		0.09
	SRMR	< 0.08		0.07
Exploratory bifactor analysis	ECV	> 0.60	Psych (R package)	0.65
	Omega *H*	> 0.80		0.78

*Abbreviations*: *CFA*, confirmatory factor analysis; *CFI*, comparative fit index; *ECV*, explained common variance; *PROMIS-PF20a*, 20-item short form of the Patient-Reported Outcomes Measurement Information System physical function item bank; *RMSEA*, root mean square error of approximation;* SF-36 PF-10*, 10-item Short Form of the Medical Outcomes Study 36 Physical Function Scale;* SRMR*, standardized root mean square residual; *TLI*, Tucker-Lewis index, weighted

Measurement invariance was supported by DIF analysis with respect to age, gender, and the clinical samples.

### Agreement of Linking Across Linking Approaches

Pearson correlations between PROMIS-PF20a and SF-36 PF-10 T-scores were consistently high (*r* ≥ 0.84) for each linking approach in the full analytic sample, as well as in each clinical sample, reflecting an initial high association between the two measures (Table 3). The mean differences of the observed and linked PROMIS-PF T-scores were ≤ 1.2 ± 4.4 for the full analytic sample and ≤ 1.6 ± 5.0 for each clinical sample, indicating only marginal score differences for all linking approaches (Table 4).

**Table 3:** Correlations, Mean Differences, and Standard Deviations of Differences Between PROMIS-PF20a T-Scores and SF-36 PF-10 T-Scores Derived from Three Linking Algorithms

	Analytic sample (*N* = 619. 100%)		Cardiology (*n* = 185. 29.89%)		Rheumatology (*n* = 172. 27.79%)		Psychosomatic (*n* = 262. 42.33%)	
	*r*	MD (SD)	*r*	MD (SD)	*r*	MD (SD)	*r*	MD (SD)
PROMIS-PF20a and SF-36 PF-10 IRT re-estimation	0.88	0.31 (4.13)	0.91	0.48 (3.29)	0.84	− 0.29 (4.97)	0.89	0.60 (4.02)
PROMIS-PF20a and SF-36 PF-10 item-level linking	0.88	− 0.11 (4.83)	0.91	− 0.17 (4.23)	0.84	− 0.10 (5.36)	0.89	− 0.07 (4.88)
PROMIS-PF20a and SF-36 PF-10 cross-walk table	0.88	− 1.19 (4.37)	0.90	− 0.98 (3.75)	0.84	− 1.62 (5.02)	0.89	− 1.05 (4.32)

*Abbreviations*: *IRT*, item response theory; *MD*, mean difference; *n*, number; *PF*, physical function; *PROMIS-PF20a*, 20-item short form of the Patient-Reported Outcomes Measurement Information System physical function item bank; *r*, correlation; *SF-36 PF-10*, 10-item Short Form of the Medical Outcomes Study 36 Physical Function Scale; *SD*, standard deviation

**Table 4:** T-Score Means and Standard Deviations of PROMIS-PF20a and SF-36 PF-10 Derived from Proposed Linking Algorithms by Analytic Sample and by Each Clinical Sample

	Analytic sample (*N* = 619. 100%)	Rheumatology sample (*n* = 172. 27.79%)	Cardiology sample (*n* = 185. 29.89%)	psychosomatic Sample (*n* = 262. 42.33%)
	Mean (SD)	Mean (SD)	Mean (SD)	Mean (SD)
PROMIS-PF20a	42.57 (8.66)	39.39 (8.98)	43.46 (7.75)	44.02 (8.55)
SF-36 PF-10 IRT re-estimation	43.26 (8.31)	40.44 (7.95)	44.01 (8.02)	44.58 (8.41)
SF-36 PF-10 Item-level linking	42.68 (10.30)	39.49 (9.78)	43.63 (9.93)	44.09 (10.48)
SF-36 PF-10 Cross-walk table	43.78 (9.15)	41.06 (8.72)	44.46 (8.68)	45.08 (9.38)

*Abbreviations*: *IRT*, item response theory; *n*, number; *PF*, physical function; *PROMIS-PF20a*, 20-item short form of the Patient-Reported Outcomes Measurement Information System physical function item bank; *SF-36 PF-10*, 10-item Short Form of the Medical Outcomes Study 36 Physical Function Scale; *SD*, standard deviation

As expected, the MAE and RMSE were lowest in the re-estimated model, indicating the most accurate estimated among the linking approaches for the observed sample at the individual level. However, taking this as the reference, the 95% CI for MAE and RMSE of the cross-walk linking overlapped with the 95% CI of the re-estimated model, indicating no significant reduction in accuracy. The results for the accuracy of item-level linking were not entirely conclusive. While the MAE indicated significant differences compared to the re-estimated model in most samples, the RMSE — being sensitive to outliers — indicated lower accuracy only in the psychosomatic sample. In sum, our results suggest somewhat lower accuracy at the individual level for the item-level linking approach compared to the reference method. However, differences between observed and linked scores can be considered of minimal practical relevance on the aggregated group level. SMDs were < 0.2 for the paired difference of each linking approach, indicating negligible effect sizes for any observed differences (Table 5).

**Table 5:** Agreement of Measures/Paired Differences After Unidimensional IRT Linking

Sample	MAE [95% CI]	RMSE [95% CI]	SMD [95% CI]
**Analytic sample**			
PROMIS-PF20a – SF-36 PF-10 IRT re-estimation	3.01 [2.89; 3.24]	4.13 [3.70; 4.64]	0.04 [0.00; 0.07]
PROMIS-PF20a – SF-36 PF-10 Item-level linking	3.61 [3.37; 3.87]	4.83 [4.37; 5.37]	− 0.01 [− 0.04; 0.03]
PROMIS-PF20a – SF-36 PF-10 Cross-walk table	3.42 [3.18; 3.67]	4.61 [4.19; 5.08]	− 0.14 [− 0.18; − 0.10]
**Cardiology sample**			
PROMIS-PF20a – SF-36 PF-10 IRT re-estimation	2.54 [2.23; 2.87]	3.32 [2.88; 3.80]	0.06 [0.00; 0.12]
PROMIS-PF20a – SF-36 PF-10 Item-level linking	3.27 [2.89; 3.66]	4.23 [3.69; 4.78]	− 0.02 [− 0.07; 0.04]
PROMIS-PF20a – SF-36 PF-10 Cross-walk table	3.10 [2.74; 3.49]	4.05 [3.54; 4.57]	− 0.13 [− 0.19; − 0.06]
**Rheumatology sample**			
PROMIS-PF20a – SF-36 PF-10 IRT re-estimation	3.37 [2.86; 3.95]	4.97 [3.73; 6.26]	− 0.03 [− 0.12; 0.05]
PROMIS-PF20a – SF-36 PF-10 Item-level linking	3.65 [3.12; 4.29]	5.34 [4.00; 6.79]	− 0.01 [− 0.10; 0.07]
PROMIS-PF20a – SF-36 PF-10 Cross-walk table	3.77 [3.23; 4.38]	5.37 [4.26; 6.57]	− 0.18 [− 0.27; − 0.10]
**Psychosomatic sample**			
PROMIS-PF20a – SF-36 PF-10 IRT re-estimation	3.12 [2.81; 3.43]	4.05 [3.67; 4.42]	0.07 [0.01; 0.13]
PROMIS-PF20a – SF-36 PF-10 Item-level linking	3.83 [3.47; 4.19]	4.88 [4.43; 5.32]	0.01 [− 0.1; 0.1]
PROMIS-PF20a – SF-36 PF-10 Cross-walk table	3.40 [3.06; 3.76]	4.43 [4.00; 4.86]	− 0.13 [− 0.19; − 0.06]

*Abbreviations*: *CI*, confidence interval; *IRT*, item response theory; *MAE*, mean absolute error; *n*, number; *PF*, physical function; *PROMIS-PF20a*, 20-item short form of the Patient-Reported Outcomes Measurement Information System physical function item bank; *RMSE*, root mean squared error; *SF-36 PF-10*, 10-item Short Form of the Medical Outcomes Study 36 Physical Function Scale; *SE*, standard error; *SMD*, standardized mean difference

**Figure. 1:**
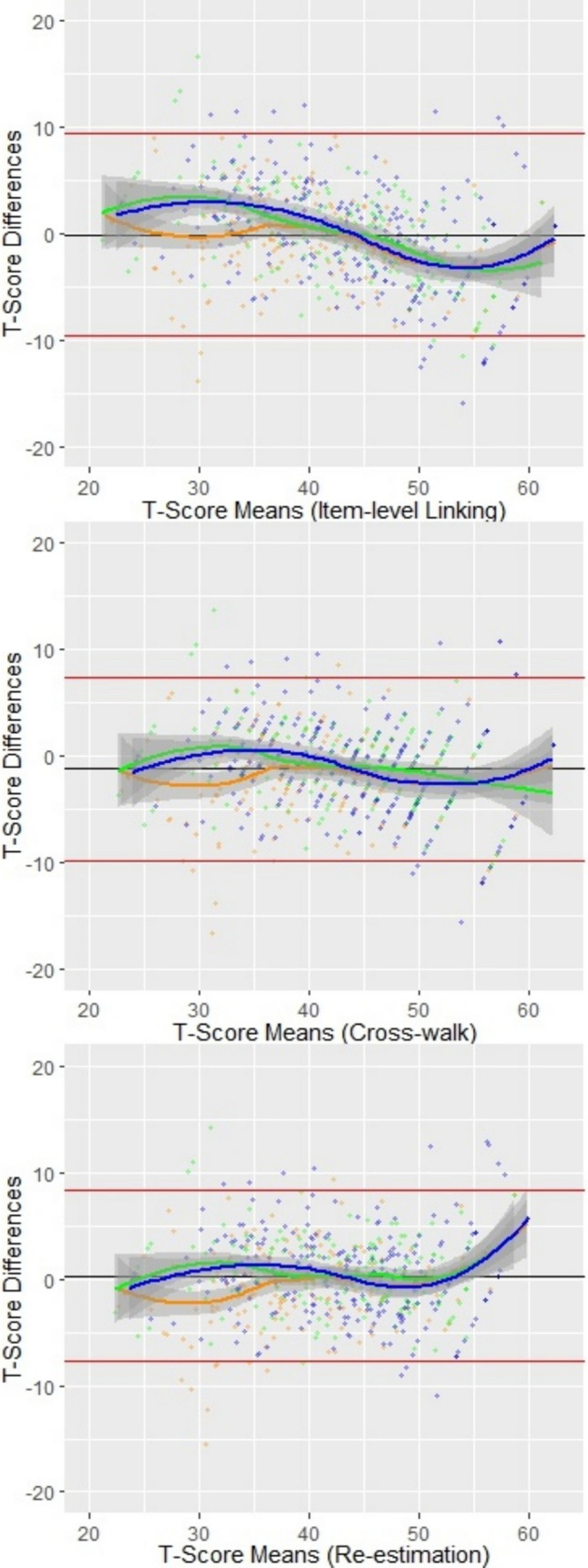
**Bland–Altman plots showing the agreement of linked T-scores from three linking algorithms for each clinical sample**. Figure Legend: The bold black line indicates the expected mean difference if there were perfect agreement. The bold red lines indicate 95% limits of agreement. The bold colored lines indicate the expected mean difference and 95% confidence interval (gray area) for each clinical sample at each average score. The orange line represents the rheumatology sample, the green line the cardiology sample, and the blue line the psychosomatic sample.

In line with these findings, Bland–Altman plots showed expected mean differences close to 0 for all linking approaches (Fig. 1). Although agreement was largely stable across the entire T-score continuum in each clinical sample, accuracy appeared to be the greatest in scores in the middle ranges of the scales. Larger differences were observed in the lower levels (T-scores < 40) and above-average levels (T-scores > 50) for all samples and for all linking approaches.

## DISCUSSION

This study was set out to evaluate two previously established approaches to link SF-36 PF-10 scores to the PROMIS-PF metric and to compare the results to those from a re-estimated model, using data from three clinical samples. The results indicate that all proposed linking methods, despite their methodological differences, yield comparably high agreement of observed and linked scores. This confirms that previously established linking approaches can be used to reliably and practically translate SF-36 PF-10 scores to standardized PROMIS-PF T-scores across different clinical samples without requiring re-estimation, supporting their broader use in clinical research.

The clinical implications of these findings are significant. The use of a standardized PRO metric, such as the PROMIS-PF, contributes to improved outcome assessment and significant scientific progress. A single common metric enables both clinicians and researchers to compare and interpret the PF level consistently, independently of the specific measure utilized. The consistency in standardized information is highly relevant in the healthcare setting, such as when analyzing outcomes across clinical trials or when comparing current and historical patient data.^17^ Moreover, the impact of using standardized metrics extends beyond clinical research, facilitating interoperability between different healthcare systems and datasets.^41^

For the translation of scores onto the standardized PROMIS-PF metric, our work proposes that user-friendly cross-walk tables may be preferred as the most practicable method. Unlike more complex IRT-based linking methods, cross-walk tables do not require statistical expertise or specialized software. Instead, they provide a straightforward tool for translation of different instruments onto the PROMIS T-score metric. Moreover, in our study, the cross-walk linking exhibited somewhat higher accuracy compared to the item-level linking, further supporting its potential as an accessible and reliable method for translating scores across different measures. Currently, the PROsetta stone offers such tables for various measures on its freely accessible website (www.prosettastone.org). Due to its accessibility and practicability, these tables can be directly embedded into workflows, including electronic health record systems, as a simple look-up Table.^42^ With this, the cost-effective potential of cross-walk tables not only facilitates score conversions, but also promises the easy implementation of the standardized PROMIS metric as the state-of-the-art PRO tool in practical settings.

As previous studies have shown, linking methods perform well at the group level, with larger sample sizes improving accuracy.^17^ However, on the individual level, there can be significant measurement error,^16,17^ and the acceptability of this error depends on the context in which the method is applied. Our study found similar results, confirming that while the linking performs very well at the group level, individual level accuracy can vary significantly, with average differences of about 3 to 4 T-scores.

Some discrepancies in the level of agreement have to be considered. While agreement was high in the score range where most participants in the sample were located, all linking approaches suggested some differences at either end of the PF-continuum. However, these are most likely a result from the limited measurement range of the SF-36 PF-10, which exhibits both floor and ceiling effects, limiting the detection of subtle variations at the extreme ends of the PF-continuum. Previous studies evaluating the relation between PROMIS-PF and SF-36 PF-10 have evidenced this issue.^17,43^ Accordingly, the SF-36 PF-10 does not cover a broad measurement range alike the PROMIS-PF, not extending much beyond the T-score mean of 50.^17^ Prior research has addressed this known problem in the PROMIS-PF domain, either by developing new floor and ceiling items^44^ or by employing extended response scales.^24^

In addition, it is important to consider how patient characteristics might affect the agreement between observed and linked scores. Although we did not evidence measurement invariance for age and gender similar to the original publication of the PROMIS-PF item bank,^16^ we found that the agreement of linking slightly deviated in the rheumatology sample in below-average PF levels. This finding is consistent with previous studies, which indicated potential disease-related DIF for cardiovascular and rheumatic patients in items assessing hand function and fine motor skill, which typically are informative at the lower end of the PF-continuum.^16,28^ Hence, when applying the linking procedure in severely functionally impaired populations, potential disease-related differences should be considered.

This study has some limitations. First, several other linking approaches exist, which were not investigated as part of the present study, such as the non-IRT approach of equipercentile linking.^30,45^ This method could potentially offer alternative insights into score agreement and might be useful in contexts where IRT assumptions do not hold. However, given the high correlation observed in our study, unidimensional IRT-based linking is the recommended approach.^17^ Second, generalization is limited to the clinical samples included in this study, as well as to the analyzed PF measures, requiring further validation studies. However, it remains that our findings provide important evidence on different linking approaches and contribute to their overall generalizability using three different clinical samples.

## Supplementary Information

Below is the link to the electronic supplementary material.

Supplementary file 1 (DOCX 65.7 KB)
