# Supplementary material for: Comparing Approaches to Link SF-36 PF-10 Scores to PROMIS Physical Function: A Validation Study in Three Clinical Samples
Source: J Gen Intern Med. 2025 Apr 29;40(14):3326–34. doi: 10.1007/s11606-025-09496-5 (PMC12586260; doi:10.1007/s11606-025-09496-5)

**Supplementary Figure**: Flow chart illustrating the derivation of PROMIS T-scores based on proposed linking methods


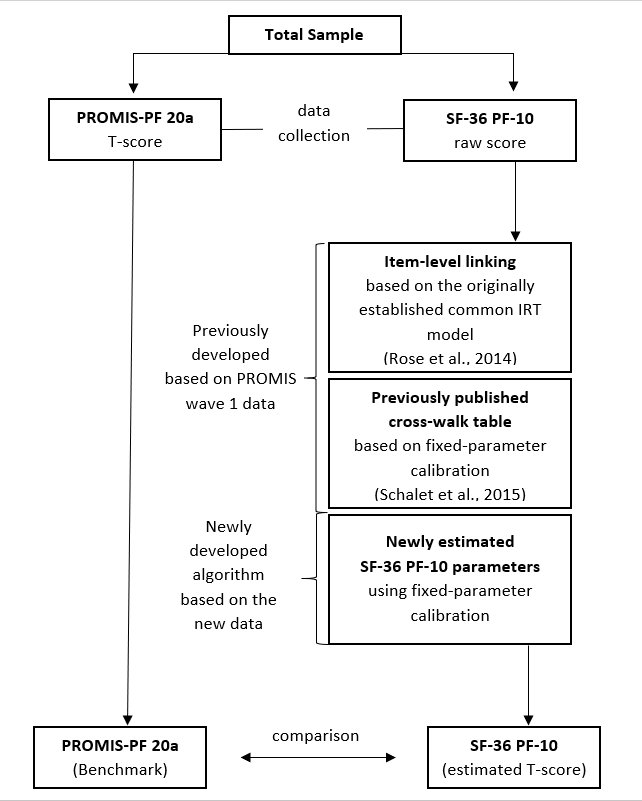

Supplement: Supplementary file 1 — (DOCX 65.7 KB) [file 11606_2025_9496_MOESM1_ESM.docx]
